# Tapping into the plasticity of plant architecture for increased stress resilience

**DOI:** 10.12688/f1000research.140649.1

**Published:** 2023-10-02

**Authors:** Maryam Rahmati Ishka, Magdalena Julkowska

**Affiliations:** 1 Boyce Thompson Institute for Plant Research, Ithaca, New York, 14853, USA

**Keywords:** plant architecture, root architecture, shoot architecture, leaf architecture, stress resilience, plant fitness

## Abstract

Plant architecture develops post-embryonically and emerges from a dialogue between the developmental signals and environmental cues. Length and branching of the vegetative and reproductive tissues were the focus of improvement of plant performance from the early days of plant breeding. Current breeding priorities are changing, as we need to prioritize plant productivity under increasingly challenging environmental conditions. While it has been widely recognized that plant architecture changes in response to the environment, its contribution to plant productivity in the changing climate remains to be fully explored. This review will summarize prior discoveries of genetic control of plant architecture traits and their effect on plant performance under environmental stress. We review new tools in phenotyping that will guide future discoveries of genes contributing to plant architecture, its plasticity, and its contributions to stress resilience. Subsequently, we provide a perspective into how integrating the study of new species, modern phenotyping techniques, and modeling can lead to discovering new genetic targets underlying the plasticity of plant architecture and stress resilience. Altogether, this review provides a new perspective on the plasticity of plant architecture and how it can be harnessed for increased performance under environmental stress.

## Introduction

Plants evolved a wide range of architectural designs, ranging from compact rosettes to sprawling vines. Selective breeding or genetic engineering has been used to alter plant architecture, creating dwarf varieties of crops that are less likely to lodge, thereby providing significant contribution to yield increase. ^1^^–^^3^ Plant architecture changes with plant development, as younger plants rely on development of the growth axis determined by the primary root and shoot meristems, while the final plant architecture depends on activation or dormancy of the axillary branches, which is controlled by internal and external signals. Every potential lateral organ is determined through cell priming into primordia, which can be dormant, aborted, or develop into new sink/source tissues. Transient environmental changes can cause changes in plant growth patterns, such as light availability throughout the day. ^4^^,^^5^ However, these changes are often reversible, and contribute to short-term stimuli responses. ^6^^,^^7^ On the other hand, long-lasting environmental changes will result in permanent changes in altered plant architecture, *e.g.*, internode elongation upon the decrease in light availability. ^8^ The absolute length of time required for permanent alterations in plant architecture will depend on the specific environmental factor, as well as developmental, genetic, and physiological contexts.

Current breeding strategies utilize the most heritable traits, with low interaction between the genotype and the environment, resulting in reliable plant performance across environmental conditions. ^9^^–^^11^ While focusing on trait stability, many components that contribute to plant performance are lost, such as interactions with the microbiome, ^12^ and stress-inducible changes in plant architecture. ^13^^,^^14^ Thus, the plant performance components that rely on phenotypic plasticity remain to be harnessed. Here, we review the concepts of plant architecture that have thus far contributed to plant productivity and stress resilience, and propose how integrating phenotypic plasticity into discovery pipelines can further expand the resilience potential of the plant.

## Aspects of plant architecture

Plant architecture describes a three-dimensional (3D) organization of plant above- and below-ground biomass between the main and lateral growth axes, through resource allocation to individual meristems. ^15^ Additionally, the activation of the vascular cambium leads to formation of secondary xylem (wood) and phloem, ^16^^–^^18^ thereby contributing to the plant width, as opposed to primary growth, which leads to an increase in the length of the plant. Both primary and secondary growth respond to internal signals and external stimuli. Within this review we divide the plant architecture into root, shoot and leaf system architecture, and describe them in the context of their contributions to environmental resilience.

### Root architecture

**Figure 1.**
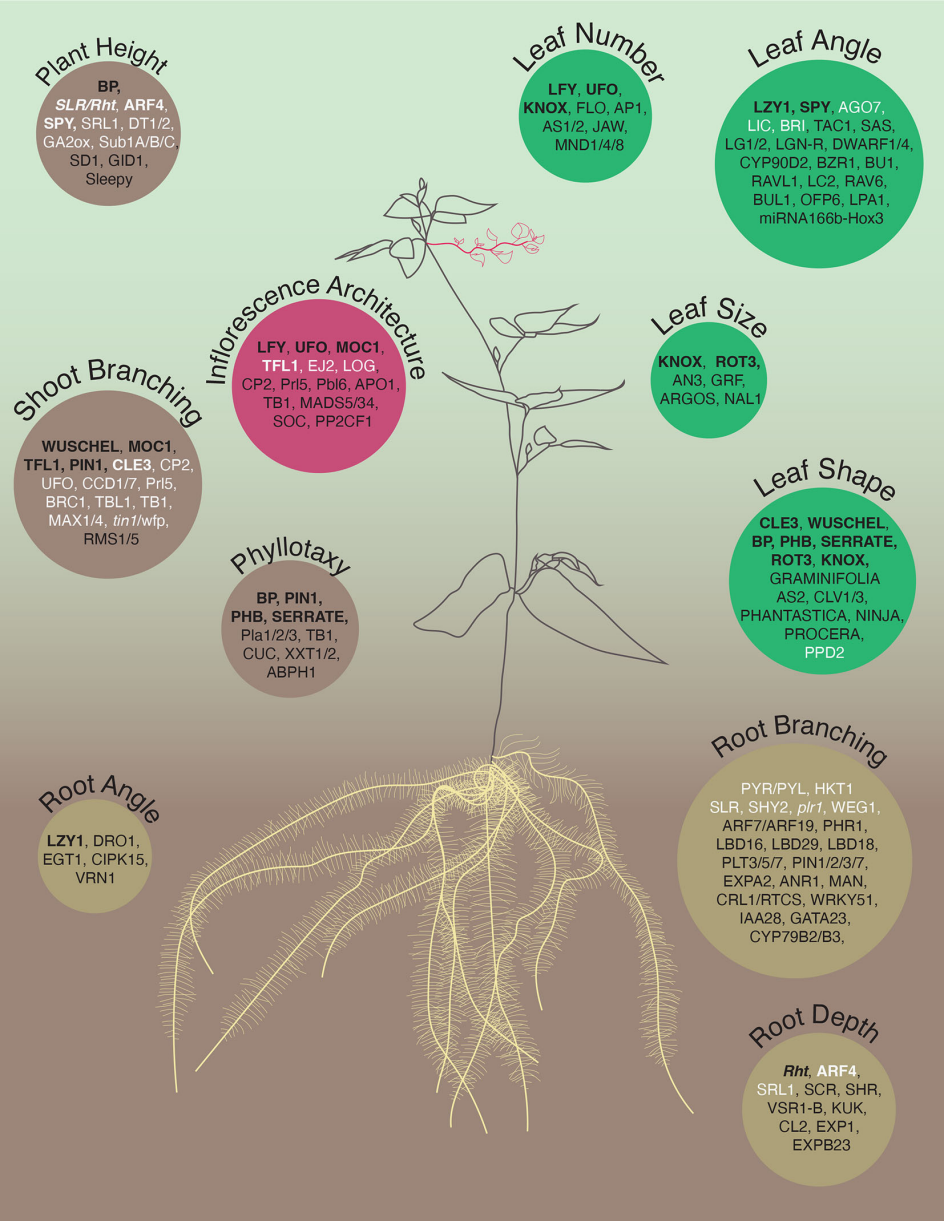
Overview of genetic components regulating plant architecture. The plant architecture determines how the biomass is distributed between primary and secondary growth axes, thereby affecting plant capacity for foraging for water and nutrients, as well as light capture. Here, we list individual genes that have been associated with components of 1) Root System Architecture: Root Depth, ^31^^,^^108^^,^^263^^–^^270^ Root Angle, ^44^^,^^45^^,^^271^^–^^277^ and Root Branching, ^57^^,^^85^^,^^89^^,^^96^^,^^265^^,^^278^^–^^298^ 2) Shoot System Architecture: Plant Height, ^138^^–^^140^^,^^266^^,^^269^^,^^299^^–^^305^ Shoot Branching, ^306^^–^^323^ Phyllotaxy, ^112^^,^^116^^,^^127^^,^^324^^–^^329^ Inflorescence Architecture, ^174^^–^^177^^,^^183^^–^^186^^,^^330^^–^^336^ and 3) Leaf System Architecture: Leaf Angle, ^148^^,^^211^^,^^212^^,^^273^^,^^337^^–^^354^ Leaf Size, ^355^^–^^360^ Leaf Shape ^306^^,^^324^^–^^326^^,^^360^^–^^369^ and Leaf Number developed prior to reproductive stage transition. ^236^^,^^327^^,^^330^^,^^367^^,^^370^^–^^373^ The genes that were observed to have pleiotropic effects on multiple components of plant architecture are listed in bold. Genes that have positive and negative effect on trait value are listed in black and white, respectively. Loci for which the causal gene has not yet been identified are listed in italics. We send our sincere apologies to researchers whose work is not included within this overview.

The root architecture encompasses the spatial configuration of the roots, including root elongation, lateral root numbers, and orientation ( Figure 1, **Table S1 in *Extended data*** ^374^). Root architecture affects a plant’s ability to absorb available water and nutrients, anchorage, and plant interactions with the soil microbiome. During the seedling establishment, the biomass investment into the main root is prioritized as it anchors the plant and determines the axis along which the soil is explored for water and nutrients. ^19^ At later developmental stages, the root architecture is dominated by lateral roots. ^20^ Lateral roots can develop into long non-determinant roots, which help with soil exploration, or shorter and thinner roots, which contribute directly to water and nutrient absorption. ^21^^,^^22^ While we are still learning to understand the fundamental differences between main roots and various kinds of lateral roots, ^23^^–^^25^ the growth of each root is determined by root apical meristem. The auxin maximum within the meristem prevents the meristem cells from differentiating and regulates cell division rates. ^26^^–^^29^ The size of the meristematic zone is affected by the environment and plant’s genetic makeup. ^30^^,^^31^ The cell elongation occurs due to the activation of plasma-membrane H ^+^-ATPases resulting in apoplast acidification. ^32^^–^^34^ Low pH activates EXPANSINs (EXP) that loosen the network of cellulose strands and other cell wall components. ^35^ The increasing pressure from the vacuole, which becomes less fragmented along the root developmental axis, results in increased turgor pressure, which contributes to cell elongation. ^36^^,^^37^

While main root tends to grow into the gravity axis, the lateral or crown roots emerge and grow initially at nearly horizontal orientation. The lateral roots reach a vertical orientation, but this “gravitropic set-point angle” (hereafter referred to as “root angle”) is determined by plant’s genetic makeup rather than gravity, and is regulated independently of the main root gravitropism. ^38^ Root angle is one of the most heritable root architecture traits. ^39^^–^^42^ Steep root angle allows for denser planting, and thus have been selected throughout the modern breeding programs. The “steep, cheap, and deep” ideotype ^43^ benefits water and nitrogen foraging. ^42^^,^^44^^–^^46^ On the other hand, shallow root systems improve foraging for less mobile nutrients, such as phosphate. ^47^ Therefore, root angle determines the overall profile of soil exploration, and impacts plant nutrient and water foraging. Additional anchoring of the plants can be also promoted through the production of adventitious and brace roots ^48^^,^^49^ that prevents the plants from being uprooted by strong winds or water flow. The root angle has been thus far predominantly explored in the monocotyledonous species in breeding programs, ^50^^–^^53^ providing better water and nutrient use efficiency, with limited or no yield penalty under non-stress conditions.

Along the growth axis of the main root, the lateral organs are initiated. The first sign of cell initiation into the lateral root primordia is the initiation of founder cells, ^54^ demarcated by the migration of cell nuclei towards each other in the two neighboring pericycle cells. The position of the founder cells follows the auxin fluctuation in the root apical meristem, ^55^^,^^56^ and relies on auxin-dependent signal transduction. ^57^^,^^58^ Auxin maximum is required to form the primordium, and surrounding cytokinin inhibits formation of new primordia. ^59^^–^^61^ The first step in differentiating the founder cells morphologically from the neighboring cells is the change in the cell division plane, from anticlinal to periclinal, resulting in a bulging structure within the root. ^62^ This change in the division plane requires repositioning of the microtubule and F-actin cytoskeleton. ^63^^–^^65^ Endogenous and exogenous factors underlying this cytoskeletal reorganization remain to be characterized. The transition from primed founder cells into lateral root primordium requires maintenance of auxin signaling ^66^^,^^67^ to establishing a new quiescent center and lateral root meristem. ^68^ Additionally, the developing lateral root primordium needs to orchestrate its clear passage through the surrounding tissues, including the endodermis and the degradation of the Casparian strip. ^69^^–^^71^ Moreover, the boundary between the proliferating and non-proliferating cells within the developing root primordium must be maintained for the successful development of new lateral roots. ^72^ The control of primordium quiescence and emergence is exerted through developmental and environmental signals that determine the final root system architecture. ^73^ The environmental signals affect lateral root primordium development at multiple points, with the majority of abiotic stresses, including hypoxia, osmotic stress, and salt stress, leading to reduced lateral root development. ^74^^–^^80^

The root architecture is determined by the distribution of biomass between the main and lateral root growth axes and the angles at which individual lateral roots develop. Nutrient deficiency, drought, and salt stress, strigolactones promote the elongation of main root growth while inhibiting the formation of lateral roots, mainly through inhibiting polar auxin transport. ^81^ Similarly, abscisic acid (ABA) reduces the growth of the main root to a lesser extent compared to the lateral root through inhibition of gibberellic acid and brassinosteroid signaling, as well as polar auxin transport, resulting in overall deeper root systems. ^82^^–^^84^ On the other hand, ethylene promotes the emergence of existing lateral root primordia while inhibiting the main root elongation, ^75^ resulting in shallower root systems. ^85^^,^^86^ Additionally, the development of individual lateral roots is also determined by the water gradients within the growth media—where lateral roots either a) continue to grow in the areas with high water availability (hydro-patterning ^87^^,^^88^), b) cease their formation when roots grow through large air-gaps in the soil (xerobranching ^89^^,^^90^). Of note, both processes seem to depend on ABA signaling. ^87^^,^^89^ The distribution of lateral root formation is also changing in response to salt stress, ^91^ nutrient availability, ^92^^,^^93^ and mechanical stimuli. ^94^^,^^95^

As root development at both architectural and anatomic levels is highly susceptible to environmental inputs, ^96^^–^^100^ it is becoming paramount to identify genetic components that contribute to root architecture development under various conditions. While the fundamental programs that guide root development and elongation have been well established, the allelic variation and genetic components that contribute to maintained root growth under adverse conditions need to be identified. Maintained root growth has been associated with increased resilience to salt stress and contributing to reduced soil erosion. ^101^^–^^104^ Additionally, combination of individual stresses often results in different root architectures compared to individual stress. ^105^ Therefore, performing more discovery-based research to identify genes controlling specific aspects of root architecture under stress conditions and their validation needs to be explored in future studies. Additionally, the aggregation of phenotypes, also known as integrative phenotypes, has been more useful in characterizing water foraging strategies between individual genotypes. The wider use of integrated phenotypes will not only diversity the possible strategies of environmental resilience, but also highlight the importance of new traits, such as penetration of hardened soils ^106^ that increases with the root diameter ^107^ and maintenance of elongation while exposed to mechanical stress. ^108^

### Shoot architecture

The shoot system architecture is determined by all the scaffolding plant structures that determine the spatial distribution of leaves, branches, and flowers ( Figure 1, **Table S2 in *Extended data*** ^374^). Shoot system architecture determines the efficiency of light capture, evapotranspiration, and the fruit-bearing capacity of the plant. Additionally, in dicotyledonous plants, the secondary growth contributes to the plant’s ability to increase the height or fruit-bearing capacity by providing additional structural support. ^109^^,^^110^ During the initial vegetative stage, the main growth axis is established by the activity of the shoot apical meristem, which produces primordia, that give rise to new leaves and lateral buds in the process called phyllotaxy. ^111^ The position of primordia is guided by the self-emergent properties of auxin ^112^^–^^114^ and cytokinin distribution. ^115^^–^^117^ High levels of auxin trigger new lateral organ initiation, ^112^^,^^118^^,^^119^ whereas cytokinin field that surrounds each auxin maximum creates an inhibitory field that prevents the formation of new lateral organs, which strength decreases with the distance. ^120^ Through a stochastic process, the first primordium develops at a random position within the shoot apical meristem, ^121^^–^^124^ giving the origin to auxin maximum and a new inhibitory cytokinin field. ^120^ The position of subsequent primordium depends on the size of the meristem, meristem growth rate, and hormone transport within the apical meristem. ^125^ The patterns of phyllotaxis are determined by the push-and-pull dynamics between auxin and cytokinin, ^116^^,^^126^ which in case of a round meristem, results in self-emergent spiral phyllotaxis, as demonstrated using the magnetic cactus experiment. ^114^ The complex geometry of the shoot apical meristem that allows phyllotactic patterns to develop relies on differential cell wall integrity, providing structural support for developing new organs as well as sufficient flexibility for cell division. This complexity in meristem geometry is accommodated by the xyloglucans, ^127^ which are responsible for the separation of the cellulose microfibrils and tethering them to other cell wall components. ^128^ Phyllotaxy has thus far not been a target in plant improvement or environmental resilience, possibly as the patterns that are present in cultivated and wild plants are already optimized for light use efficiency through reduced self-shading. ^129^

The plant height contributes to plant performance, as reducing plant height provides more resources to be allocated to the inflorescence and final yield. The “Green Revolution” targeted gibberellin biosynthesis and sensitivity, ^130^ thereby increasing nitrogen assimilation and allocation of assimilates to the grain. ^131^ While high plants might produce increased yield due to increased access to light, this requires additional mechanical support, that can be provided by secondary growth. However, as secondary growth is absent in monocotyledonous plants that constitute the majority of our staple crops, shorter plant statues are a desirable agronomic trait. The plant height is an ideal breeding target, as the heritability is high, it significantly contributes to lodging resistance, facilitates denser cultivation, and easy manual and mechanical harvest. ^132^^–^^135^ While many stresses decrease plant height, ^136^^,^^137^ it is still undetermined whether this phenomenon is due to an overall decrease in plant growth rate, or rather active reprogramming of plant architecture and investment into lateral branches. One clear example where limiting plant height is beneficial is Sub1 locus in rice, ^138^^,^^139^ containing three ethylene response factors (SUB1A, SUB1B, SUB1C, **Table S2 in *Extended data*** ^374^). In the event of flooding, Sub1 locus restricts ethylene production and gibberellin responsiveness, which effectively arrests the growth. ^140^ This allows the plant to endure submergence and resume growth once floodwaters recede, without any yield penalty under non-flooded conditions.

Apical dominance results in height gain at the expense of lateral growth by inhibiting the auxiliary buds. Similar to plant height, reduced branching is an important target in plant improvement and domestication, as it redirects the assimilates to the fruit and grain, and limits the resource investment into the vegetative phase to the absolute minimum. The advantage of apical dominance or highly branched shoot architecture depends on the species and environment. In competitive environments, such as densely cultivated plots in our current agriculture, vertical growth is necessary to ensure access to light, and thus apical dominance provides an advantage. ^141^^,^^142^ For perennial species in low competitive environments, increased branching results in higher levels of source tissues, as the number of leaves that sustain plant growth and fruit production increase with each lateral branch. ^143^ Environmental signals of light quality and quantity, ^144^^–^^149^ as well as water and nutrient availability ^150^^,^^151^ were previously described to modulate branching, presumably to balance the respiratory and nutritional demands of leaves in limiting conditions. Apical dominance is maintained by dormancy of auxiliary buds, which requires high levels of strigolactones and auxin, and low levels of cytokinin, ^152^^–^^155^ and is released by high availability of sugars. ^156^^–^^158^ Increased sugar availability promotes cytokinin biosynthesis, while reducing the strigolactone signaling. ^157^^,^^159^^,^^160^ During the dormancy release, the sugar availability is also promoting the local auxin biosynthesis, which in turn activates the gibberellins. ^161^ Sustained bud outgrowth is dependent on continued sugar supply and auxin-dependent production of gibberellins, which promotes the sink strength of the developing bud, thereby ensuring continued bud development into a new branch. ^161^ Brassinosteroids also play an important role in the release of apical dominance, through release of the transcription factor (BZR1, **Table** **S2 in *Extended data*** ^374^), that suppresses the expression of BRANCHED1 (BRC1), an inhibitor of bud outgrowth. ^162^ Stress-induced repression of branching is orchestrated through ABA, which acts downstream of the BRC1. ^163^ The plasticity of branching was significantly reduced during domestication, ^164^^,^^165^ allowing for more space-efficient high-density planting and less labor-intensive agronomic practices. The manipulation of strigolactone-related genes is also used to enhance domestication characteristics—such as suppressed branching and tillering. ^166^^,^^167^ Although apical dominance is agronomically desirable trait, it is a risky strategy from the environmental resilience perspective, as all the resources are invested into one main growth axis, rather than “spreading the risk” with the indeterminate growth form. As apical dominance limits vegetative biomass, it might limit yield potential, as recently demonstrated in the case of buckwheat. ^168^ While apical dominance is highly preferred by modern agriculture, it is possible that improving resilience of our food production systems might require rethinking of this strategy.

The inflorescence architecture determines the arrangement of flowers on the plant and is key determinant of plant reproductive success. Inflorescence architecture is determined by the initiation of the inflorescence meristem, developing a branching pattern within the inflorescence meristem, and initiation of floral meristems. ^169^ Some inflorescence are determinate—giving rise to only one flower—whereas others undergo a number of rounds of inflorescence branching prior to developing terminal flowers. Similarly to shoot branching, this process is balanced through interactions between auxin, cytokinin, and sugar signaling. ^170^^–^^172^ Various architectures of inflorescence can be explained using a simple model, ^173^ where LEAFY/FALSIFLORA gene ^174^^–^^176^ determines the branching capacity of the inflorescence meristem, while TERMINAL FLOWER 1/SELF-PRUNING gene is regulating the timing where branches develop into determinant floral meristems. ^177^^–^^182^ While excessive branching leads to many small fruits that undergo developmental arrest, ^183^ plant breeding programs focus on finding the Goldilocks ratio between branching and determinate growth, which in most species are regulated by MADS-box transcription factors. ^185^^–^^189^ Inflorescence architecture is highly affected by environment, which significantly contributes to yield gap under adverse conditions. ^190^^–^^192^ Identification of molecular factors that affect inflorescence development under stress conditions, as well as exploring environmentally sensitive aspects of cytokinin, auxin and sugar signaling, can lead to increased environmental resilience and significant alleviation of the yield penalty. ^193^^–^^196^

### Leaf architecture

Leaf system architecture constitutes the specific characteristics of the leaves, such as size, shape and angle, as well as the number of leaves that the plants produce prior to initiation of reproductive phase ( Figure 1, **Table S3 in *Extended data*** ^374^). These aspects differ from each other in their plasticity and response to the environment. While leaf shape is determined predominantly by the developmental cues, leaf angle and leaf number prior to flowering are highly influenced by the environment. ^197^^–^^200^ Leaf system architecture plays an important role in crop productivity, as it impacts light capture, photosynthesis and transpiration rates. ^201^^,^^202^ While leaf angle can maximize the light capture and contribute to maximizing the photosynthetic rate, ^7^ desert plants are known to orient their leaves to minimize direct exposure to sunlight, thereby minimizing water loss. ^203^ The leaf angle is typically determined by the structure and development of petiole or pulvinus cells in dicotyledonous plants, while in monocotyledonous plants the leaf angle is determined between the degree of bending between the leaf sheath and leaf blade. ^199^^,^^204^^,^^205^ A pulvinus is a specialized structure located at the base of leaf blade in certain plants (grape, legumes, citrus), functioning as a joint between leaf and stem. ^206^^–^^209^ The petiole constitutes the stalk that attaches the leaf blade to the stem, also plays a critical role in determining leaf angle. Variations in cell elongation and thickness across all of these structures affect leaf angle ^199^ through auxin and brassinosteroid signaling that control ion fluxes ^207^^,^^210^ and cell wall stiffness ^211^^,^^212^( Figure 1, **Table S3 in *Extended data*** ^374^). Maintained flexibility in leaf angle has been hypothesized to be an adaptive trait under high temperature, drought, salt and flood conditions. ^199^^,^^213^

Another trait that seems to be highly adaptive to the environment is leaf size. Plants that are native to arid areas typically develop smaller or narrower leaves compared to their relatives from wetter regions. ^214^^–^^217^ Similar to other lateral organs, the leaf size is controlled by coordination of cell division and expansion, regulated by auxin and cytokinin, as well as their interactions with other hormones, that were reviewed elsewhere. ^218^^–^^220^ Overall, the increase in leaf area index (ratio between the area of one leaf to plant ground coverage) leads to more efficient light capture up to a certain point, after which self-shading reduced the plant’s overall light use efficiency. ^221^^,^^222^ As reduction of leaf size is typically associated with reduced overall light capture and yield, and is not exhibiting high phenotypic plasticity, it has thus far not been utilized in breeding programs.

Similarly to leaf size, the leaf shape is a highly heritable trait that is mainly controlled by the developmental program of the plant, ^223^^,^^224^ albeit it is receptive in some species to light and sugar availability. ^198^ The differences in leaf shape are due to development of serrations, lobes or secondary leaflets during its development. ^224^ The more complex leaf shapes are caused by re-initiation of auxin maxima within the developing leaf, resulting in cell dedifferentiation, maintained by genes involved in auxin and cytokinin signaling ( **Table S3 in *Extended data*** ^374^). Narrower leaves tend to be more common in the plants adapted to climates with extreme temperatures and low freshwater availability, whereas species with highly dissected leaves show higher rates of carbon gain and water loss compared to species with less-dissected leaves. ^225^^–^^227^ Although many molecular markers were identified to underlie variation in leaf shape, they are yet to be used as targets in plant breeding, and further evaluation between leaf shape, plant productivity and stress resilience. ^228^^–^^230^ Future studies are necessary to identify not only specific leaf shapes as well as integrated leaf shape and anatomy phenotypes (set of characteristics) that provide more insight into the relationship between optimizing light and carbon capture, while minimizing water loss and heat damage.

The number of leaves produced by a plant prior to initiation of inflorescence meristem determines the duration of the vegetative growth phase, and thus the total photosynthate that can be produced and turned into yield. In the changing climate, shortening the vegetative growth is becoming more desired to either a) escape the most severe conditions during the growth season or b) spread the risk between two or three growth cycles in more tropical regions. ^231^^,^^232^ The transition from vegetative to reproductive stage is typically regulated by a combination of developmental and environmental signals, and is currently explored through the speed breeding approach. ^233^^,^^234^ The alternative to shortened life cycle would be an indeterminate growth that is exhibited by many wild crop relatives that flower soon after germination and continue to produce flowers and fruit until the resources run out. ^235^^,^^236^ While an increase in indeterminate growth might help spread the environmental risks, this approach would require tremendous efforts in adopting the modern agronomic systems for indeterminate growth. Additionally, the wild indeterminate growth of wild crop relatives would have to be tamed by optimizing the crop’s architecture and re-allocation of photosynthate and nutrients into developing fruits.

## Challenges and future directions

As discussed above, many components of plant architecture show plasticity in response to environment and the fundamental processes underlying development of plant architecture in major plant species (Arabidopsis, tomato, maize, rice, **Tables S1-S3 in *Extended data*** ^374^) are well characterized. However, identification of loci contributing to plant architecture either constitutively, or in response to environment, in wider diversity of crops remains to be identified. Previous studies suggest that inducible controls of plant architecture, such as SUB1A or HKT1 ( **Tables S2 and S1 in *Extended data*** ^374^) provide improved environmental resilience without the trade-offs under non-stress conditions. Additionally, harnessing the potential of new discoveries by high-throughput phenotyping and molecular technologies, will provide improved understanding of nuanced control of plant architecture and functional relationships, and their contextual dependencies, between plant architecture and anatomy components and environmental resilience.

### Identify modules regulating plant architecture across plant species

Current studies of plant architecture focus on a handful of species that are either of high economic importance or have been developed as model species throughout the years for the specific questions. However, the genetic information within one species is not providing exhaustive representation of the mechanisms that contribute to plant productivity and environmental resilience across the plant kingdom. Improved understanding of fine-tune points of developmental mechanisms will provide more reliable selection of breeding targets and increased likelihood that selected alleles will provide improved performance across wide variety of elite varieties. This can be achieved through comparative genomics and phylogenetic analyses of genes that were previously identified to be involved in regulating plant architecture, and their functional characterization. ^237^^–^^239^

Improving genomic resources for wild crop relatives and indigenous crops provide better resources for development of diversity panels and mapping populations. ^240^^–^^243^ Forward genetic studies, such as genome-wide association studies (GWAS) and quantitative trait locus (QTL) mapping, combined with more accessible plant phenotyping, ^244^^–^^246^ will help to identify new genetic regions associated with changes in plant architecture, productivity and stress resilience. As in-depth phenotyping will provide more insight into timing of plant architecture development, we will be able to predict the tissues and time points that undergo transcriptional and/or metabolic reprogramming with increasing precision. Targeting specific tissues and time points for omics analyses will further reveal the molecular networks underlying regulation of plant architecture and its plasticity in response to environment. ^247^

### Determining developmental stages impacting plant architecture

As plant architecture focus until now has been predominantly to study the highly heritable components of architecture, the loci that determine the plant architecture plasticity remain to be identified. And thus, it is important to identify the specific developmental stages at which changes in plant architecture have the highest contribution to plant performance and stress resilience. ^248^ Controlled environment experiments, while they simplify the environmental responses, can provide systematic exposure to altered conditions at various developmental stages. ^249^^,^^250^ Combining controlled environment stress exposure with high-throughput phenotyping, can provide insight into how specific stress applications affect plant growth and development over time, thereby identifying critical growth stages and architectural traits that contribute to plant performance and environmental resilience. ^251^ Increasing the throughput of screening large amounts of plants and automated extraction of traits of interest will unlock the opportunities for genomic and genetic approaches that typically require large population screenings.

Together, these new developments will improve our general understanding of plant physiology and inform the expansion of future modeling approaches. Identification of key developmental stages for individual aspects of architecture will allow optimal timing for application of treatments and agronomic interventions, such as application of fertilizer, pesticide or additional irrigation. Additionally, we can focus our breeding programs or gene expression studies focusing on specific developmental stages where the specific aspects of architecture are being determined. Moreover, understanding how plant architecture develops throughout the plant lifecycle will provide insights into how different growth stages interact and contribute to the overall performance and stress resilience of the plant. Understanding how stress susceptibility changes with plant developmental stages can guide the timing of interventions to improve resilience and enhance the accuracy of predictive crop growth models.

### Quantifying plant architecture beyond canonical traits

Current methods for quantifying plant architecture are still mainly relying on the manual characterization of plant characteristics taken at one-time point. ^245^ While we can learn a lot from counting the number of leaves, branches and roots, there is more to plant architecture than a static image of traits that are easily scored. Quantifying plant architecture beyond canonical traits involves understanding the functional and structural components of the plants, and incorporating plant architecture plasticity in response to time and the environment.

The complementation of high-throughput phenotyping methods with mathematical models allows for a more comprehensive understanding and characterization of plant architecture, including its trajectory through the dimensions of time and environment. The mathematical models can be classified as either “Growth and Development” or “Functional-Structural Plant” models. The growth and development models are typically simulating the progress of plant growth and development based on physiological factors, and provide how plant architecture changes over time. ^252^ Examples of growth and development models range from simple linear growth models to estimate growth of individual organs ^100^ to Lindenmeyer-system (L-system) models, where the growth of the entire plant is summarized by the set of context-free grammar rules. ^253^ The functional-structural plant models, on the other hand, combine plant structure with physiological processes, such as photosynthesis, respiration, and transpiration. ^254^ Examples of functional-structural plant models include models of water and ion uptake ^255^^,^^256^ and estimations of plant architecture efficiency concerning transportation cost and speed trade-offs. ^257^^,^^258^

The limitations of current models include that they often consider only a very specific developmental stage or tissue, and allow very limited input from the environment. While such models can provide tremendous insight, they often rely on plant architectural or anatomical data that can be collected only using semi-automated methods. ^259^^–^^261^ These quantification methods require substantial user input for model corrections, and thus are not suitable for large population screens. Additionally, many 3D reconstruction tools are limited to the root systems, where individual roots can be distinguished, but does not produce suitable images for shoot or inflorescence architecture, where foliage/fruit obstructs the insight into the branching structures. The implementation of technologies such as X-ray imaging could provide additional push towards simplifying the data necessary for such models. ^262^

## Conclusions

As described above, plant architecture provides significant contributions to plant productivity and stress resilience, with multiple components showing varying levels of plasticity in response to changing environment. Tapping into this plasticity, by performing discovery-based studies across various environmental conditions and wider variety of species, will provide better understanding of plant development, as well as improved selection of breeding targets for improved resilience. Automated imaging and modification of environmental aspects under controlled conditions will provide better understanding of specific developmental stages that are crucial for development of specific aspects of plant architecture. Combining high-throughput phenotyping with mathematical modeling will not only provide better noise-to-signal ratio in forward genetic studies, but will also provide improved understanding of functional aspects of plant architecture. Finally, including more species in the study of resilience and plant architecture will provide more holistic understanding of genetic and molecular modules responsible for fine-tuning plant architecture for improved resilience and ensuring food security for various agronomic and cultural regions.

## Data Availability

No data are associated with this article. Figshare: Genes and genetic loci involved in regulation of plant architecture. https://doi.org/10.6084/m9.figshare.24018564. ^374^ This project contains the following extended data:
‐
Table S1 (root architecture) ‐
Table S2 (shoot architecture) ‐
Table S3 (leaf architecture) Data are available under the terms of the Creative Commons Zero “No rights reserved” data waiver (CC0 1.0 Public domain dedication).
