# Supplementary material for: Tapping into the plasticity of plant architecture for increased stress resilience
Source: F1000Res. 2023 Oct 2;12:1257. [Version 1] doi: 10.12688/f1000research.140649.1 (PMC10905174; doi:10.12688/f1000research.140649.1)
Supplement: Peer review history

# Reviewer report: Reviewer response for version 1

**DOI:** 10.5256/f1000research.154027.r245280

**Published:** 2024-02-23

**Authors:** Sadikshya Poudel

**Affiliations:** 1 Washington State University, Pullman, Washington, USA

**Reviewer comments**

 The review paper is well-written and easy to understand. The authors have done an excellent job of explaining the fundamentals of plant architecture and its plasticity under various stress conditions. The detailed explanation of the root, shoot, and leaf systems is particularly commendable, providing a comprehensive understanding of how plants adapt to their environment. Additionally, the authors have effectively discussed the genetic components that regulate plant architecture, supplementing their explanations with a self-explanatory figure, which enhances the clarity and accessibility of the content. Furthermore, the paper adeptly addresses the challenges and future directions in an easily understandable manner, which is essential for the readers. By outlining the obstacles and potential paths forward, the authors have not only provided a valuable summary of the current state of research but have also offered a roadmap for future investigations in this critical area of study. This forward-looking approach adds significant value to the paper, making it a relevant and insightful resource for both researchers and practitioners in the field of plant biology and agriculture.

**Comment:** Page 5, second paragraph; *Nutrient deficiency, drought, and salt stress, strigolactones promote the elongation of main root growth while inhibiting the formation of lateral roots, mainly through inhibiting polar auxin transport.*

 This line needs to be edited. “ *Under* *nutrient deficiency, drought…...”*

**Comment: **Page 5, third paragraph; “ *The wider use of integrated phenotypes will not only diversity the possible strategies of environmental resilience…”*

 Does the author mean to diversify? Please edit the line as needed.

**Comment: **Page 5, last paragraph; *“High levels of auxin trigger new lateral organ initiation whereas cytokinin field that surrounds each auxin maximum creates an inhibitory field that prevents the formation of new lateral organs, which strength decreases with the distance.”*

 This sentence is complex to understand. Please check the structure of the sentence and make it simpler. Please split the sentence into two to make it simpler and more understandable.

**Comment: **Page 6, second paragraph; *“While high plants might produce increased yield due to increased access to light, this requires additional mechanical...”*

 Here does the author refer to taller plants by mentioning high plants? I suggest using the term tall or taller plants instead of high plants if you are referring to height.

**Comment:** Page 6. The author has mentioned the term *strigolactone* multiple times. I suggest adding a brief line about this term in the paper.

**Comment**: Page 6. I suggest the author explain the term Goldilocks ratio for a better understanding to a layman.

**Comment:** Page 7. *“Plants that are native to arid areas typically develop smaller or narrower leaves compared to their relatives from wetter regions.” *I suggest the authors add literature on why the leaves are narrower in arid natives.

**Comment:** The author has provided a comprehensive explanation of plant architecture; however, there appears to be limited mention of plasticity and how it changes under different stress conditions. I suggest that the authors incorporate references to additional studies highlighting the plasticity of various crops in response to diverse stressors. Acknowledging and integrating a wider range of research in this area can enhance the depth of the discussion and contribute to a more comprehensive understanding of crop resilience.

Is the review written in accessible language?

Yes

Are all factual statements correct and adequately supported by citations?

Yes

Are the conclusions drawn appropriate in the context of the current research literature?

Yes

Is the topic of the review discussed comprehensively in the context of the current literature?

Yes

Reviewer Expertise:

Plant stress physiology, barley breeding, biofortification

I confirm that I have read this submission and believe that I have an appropriate level of expertise to confirm that it is of an acceptable scientific standard.

# Reviewer report: Reviewer response for version 1

**DOI:** 10.5256/f1000research.154027.r224113

**Published:** 2024-02-21

**Authors:** Adriana Garay Arroyo

**Affiliations:** 1 Laboratorio de Genética Molecular, Desarrollo y Evolución de Plantas, Depto. de Ecología Funcional. Instituto de Ecología, Universidad Nacional Autonoma de Mexico, Mexico City, Mexico City, Mexico

The manuscript “Tapping into the plasticity of plant architecture for increased stress resilience” by Maryam Rahmati Ishka and Magdalena Julkowska has important and accurate information in each of its sections. It is an interesting review that will be useful for the plant development community.

 Some comments: in bold are some phrases written by the authors and I wrote below the phrase, some comments on them.

**Root architecture:**

- “ **Selective breeding or genetic engineering has been used to alter plant architecture, creating dwarf varieties of crops that are less likely to lodge, thereby providing significant contribution to yield increase**”
- It could be interesting to put more examples of using selective breeding or genetic engineering as it seems that this was the only goal of these kind of studies.
- “ **while the final plant architecture depends on activation or dormancy of the axillary branches, which is controlled by internal and external signals**”
- Also, the primary root and shoot meristems depends on internal and external signals, you said this later in the text, but I suggest that you put this paragraph here: “ **Both primary and secondary growth respond to internal signals and external stimuli**”.
- “ **The absolute length of time required for permanent alterations in plant architecture will depend on the specific environmental factor, as well as developmental, genetic, and physiological contexts**”
- This is a very relevant information, please put references. It would be very interesting to know how this length is mechanistically determined.
- “ **Current breeding strategies utilize the most heritable traits, with low interaction between the genotype and the environment, resulting in reliable plant performance across environmental conditions**”
- This phrase seems contradictory, the breeding strategies are meant to be useful in specific environments and not across different environmental conditions. Please comment on this.
- “ **Lateral roots can develop into long non-determinant roots, which help with soil exploration, or shorter and thinner roots, which contribute directly to water and nutrient absorption**”
- Many lateral roots display contrasting development in homogeneous growth conditions, a phenomenon that has been called developmental instability (Muller et al., 2019). Please comment on this.
- **Figure 1**. There are more genes that participate in root depth: *XAL1* ref [4], *XAL2* ref [3] and also, *ANR1* ref [1], ref [2].
- “ **Nutrient deficiency, drought, and salt stress, strigolactones promote the elongation of main root growth**”
- I do not completely understand this phrase.
- I think it would be important to talk in general about the participation of the phytohormones in the control of development in general.
- “ **when roots grow through large air-gaps in the soil (xerobranching). Of note, both processes seem to depend on ABA signaling**”
- The second one depends also on ethylene and auxin (Bennett M).
- “ **combination of individual stresses often results in different root architectures compared to individual stress**”
- Use more than one stress under controlled conditions? This looks like a proposal to me that can be incorporated in the Conclusions section.

**Shoot architecture**

- “ **Through a stochastic process, the first primordium develops at a random position within the shoot apical meristem**”
- It will be important to consider the stochasticity in modelling, please comment on this.
- “ **and leaf number prior to flowering are highly influenced by the environment**”
- and after flowering?
- “ **However, the genetic information within one species is not providing exhaustive representation of the mechanisms that contribute to plant productivity and environmental resilience across the plant kingdom**” and in the same paragraph “ **This can be achieved through comparative genomics and phylogenetic analyses of genes that were previously identified to be involved in regulating plant architecture, and their functional characterization**”. Seems contradictory to me, the genes important in one species might be important in another.

**Conclusions**.

 These two conclusions could be together “ **including more species in the study of resilience and plant architecture will provide more holistic understanding**”  “ **by performing discovery-based studies across various environmental conditions and wider variety of species, will provide better understanding of plant development, as well as improved selection of breeding targets for improved resilience**”

 Another conclusion could be: Using high-throughput phenotyping with genome approaches.

 I suggest making a Table with the comparisons of the most important data on breeding programs and genetic engineering, that has been done in root, shoot and leaves.

Is the review written in accessible language?

Yes

Are all factual statements correct and adequately supported by citations?

Yes

Are the conclusions drawn appropriate in the context of the current research literature?

Yes

Is the topic of the review discussed comprehensively in the context of the current literature?

Yes

Reviewer Expertise:

Develpmental biology

I confirm that I have read this submission and believe that I have an appropriate level of expertise to confirm that it is of an acceptable scientific standard, however I have significant reservations, as outlined above.

## References

[1] : MADS-box transcription factor OsMADS25 regulates root development through affection of nitrate accumulation in rice. *PLoS One* .2015;10(8) : 10.1371/journal.pone.0135196 e0135196 10.1371/journal.pone.0135196 26258667 PMC4530940

[2] : MADS-box genes underground becoming mainstream: plant root developmental mechanisms. *New Phytol* .2019;223(3) : 10.1111/nph.15793 1143-1158 10.1111/nph.15793 30883818

[3] : The MADS transcription factor XAL2/AGL14 modulates auxin transport during Arabidopsis root development by regulating PIN expression. *EMBO J* .2013;32(21) : 10.1038/emboj.2013.216 2884-95 10.1038/emboj.2013.216 24121311 PMC3817466

[4] : An AGAMOUS-related MADS-box gene, XAL1 (AGL12), regulates root meristem cell proliferation and flowering transition in Arabidopsis. *Plant Physiol* .2008;146(3) : 10.1104/pp.107.108647 1182-92 10.1104/pp.107.108647 18203871 PMC2259045

# Reviewer report: Reviewer response for version 1

**DOI:** 10.5256/f1000research.154027.r234668

**Published:** 2024-02-09

**Authors:** Rumyana Karlova

**Affiliations:** 1 Laboratory of Plant Physiology, Wageningen University & Research, Wageningen, Gelderland, The Netherlands

This is a very well written review paper describing genetic control of plant architecture traits (root and shoot architecture) and their effect on plant performance under environmental stress. There is a very useful Figure 1 summarizing the molecular regulators of the plant architecture as well as 3 supplemental figures! This review is very relevant and provides further understanding of genetic and molecular regulation of plant architecture for improved crop resilience in the changing climate.

Is the review written in accessible language?

Yes

Are all factual statements correct and adequately supported by citations?

Yes

Are the conclusions drawn appropriate in the context of the current research literature?

Yes

Is the topic of the review discussed comprehensively in the context of the current literature?

Yes

Reviewer Expertise:

Plant physiology

I confirm that I have read this submission and believe that I have an appropriate level of expertise to confirm that it is of an acceptable scientific standard.
